# Investigation of a SARS-CoV-2 B.1.1.529 (Omicron) Variant Cluster — Nebraska, November–December 2021

**DOI:** 10.15585/mmwr.mm705152e3

**Published:** 2021-12-31

**Authors:** Lauren Jansen, Bryan Tegomoh, Kate Lange, Kimberly Showalter, Jon Figliomeni, Baha Abdalhamid, Peter C. Iwen, Joseph Fauver, Bryan Buss, Matthew Donahue

**Affiliations:** ^1^Nebraska Department of Health and Human Services; ^2^Epidemic Intelligence Service, CDC; ^3^CDC Foundation, Atlanta, Georgia; ^4^Public Health Solutions District Health Department, Crete, Nebraska; ^5^Nebraska Public Health Laboratory, Omaha, Nebraska; ^6^College of Public Health, University of Nebraska Medical Center, Omaha, Nebraska; ^7^Division of State and Local Readiness, Center for Preparedness and Response, CDC.

The B.1.1.529 (Omicron) variant of SARS-CoV-2 (the virus that causes COVID-19) was first detected in specimens collected on November 11, 2021, in Botswana and on November 14 in South Africa;* the first confirmed case of Omicron in the United States was identified in California on December 1, 2021 (*1*). On November 29, the Nebraska Department of Health and Human Services was notified of six probable cases^†^ of COVID-19 in one household, including one case in a man aged 48 years (the index patient) who had recently returned from Nigeria. Given the patient’s travel history, Omicron infection was suspected. Specimens from all six persons in the household tested positive for SARS-CoV-2 by reverse transcription–polymerase chain reaction (RT-PCR) testing on December 1, and the following day genomic sequencing by the Nebraska Public Health Laboratory identified an identical Omicron genotype from each specimen (Figure). Phylogenetic analysis was conducted to determine if this cluster represented an independent introduction of Omicron into the United States, and a detailed epidemiologic investigation was conducted. This activity was reviewed by CDC and was conducted consistent with applicable federal law and CDC policy.^§^

**FIGURE:**
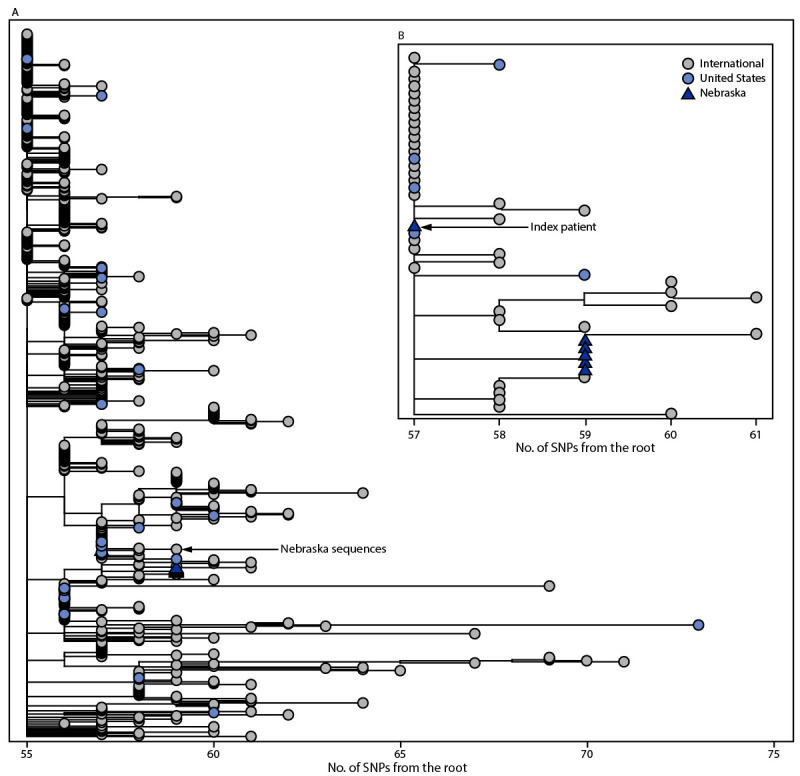
Global phylogeny of B.1.1.529 (Omicron) samples available on Global Initiative on Sharing All Influenza Data* as of December 6, 2021 (650 total genomes) (A) and expanded view of Omicron sequences^†^ (B) — Nebraska, November–December 2021^§^^,^^¶^^,^**^,^^††^^,^^§§ ^ **Abbreviation**: SNP = single nucleotide polymorphism. * https://www.gisaid.org ^†^ Branch lengths are shown in number of mutations from the root. The maximum-likelihood phylogenetic trees are rooted with the original SARS-CoV-2 genome Wuhan/Hu-1/2019. ^§^ Each of the six SARS-CoV-2 genomes generated from this cluster is >94% complete and shares 100% nucleotide identity across the length of the genome, consistent with household transmission. ^¶^ Genomes from the five secondary cases have SNPs at nucleotide positions T1552C and C23709T that are not yet found in other Omicron genomes sampled. ** The genome from the index patient, NCOV21-42615, has the ambiguous nucleotide “N” at positions 1552 and 23709 and further inspection of the read-level data showed nucleotide variability at both sites. The SNP allele frequency at these sites in the NCOV21-42615 genome is >50%, consistent with epidemiologic findings of household transmission from the index patient to all secondary cases. ^††^ https://academic.oup.com/bioinformatics/article/34/23/4121/5001388 ^§§^ https://onlinelibrary.wiley.com/doi/full/10.1111/2041-210X.12628

The index patient, who was unvaccinated, had a history of domestically acquired symptomatic SARS-CoV-2 infection confirmed by RT-PCR a year prior in November 2020. He reported unmasked close contact^¶^ with a masked, coughing person on November 20, 2021, during an international conference in Nigeria, which included attendees from multiple African countries. Before his return trip to the United States, he completed required pretravel testing with receipt of a negative antigen test result on November 21. Upon his return on November 23, while still asymptomatic, he had unmasked close contact with five household contacts. One household contact was fully vaccinated** (second Pfizer-BioNTech vaccine dose received in August 2021) and had previous symptomatic COVID-19 (RT-PCR confirmed in November 2020), three were unvaccinated and had previous symptomatic COVID-19 (RT-PCR confirmed in November 2020), and one was unvaccinated and had mild upper respiratory symptoms in November 2020, just before illness onset in the other household members, but received a negative SARS-CoV-2 RT-PCR test result at that time. No household members reported underlying medical conditions or immunocompromising conditions known to increase the risk for severe COVID-19 or diminish response to vaccination.^††^

On November 24, 2021, the index patient experienced symptoms consistent with COVID-19^§§^ and initially received a positive SARS-CoV-2 antigen test result from a local medical center on November 26. All six household members (median age = 18.5 years; range = 11–48 years) experienced symptom onset during November 24–26; median interval between earliest possible exposure to the index patient and symptom onset was 73 hours (range = 33–75 hours). The index patient and the four household contacts with previous confirmed infections described the symptoms and severity of their recent COVID-19 infection as being similar to or milder than those during their first infection. The five reinfected patients experienced fewer current symptoms, including loss of taste (none), loss of smell (none), and subjective fever (two), compared with symptoms reported during their first infections (four, four, and four, respectively). The unvaccinated patient without a previous COVID-19 diagnosis experienced cough, joint pain, congestion, fever, and chills. None required hospitalization for either their first or second infections. Twelve close community contacts of the family were identified. Four consented to testing for SARS-CoV-2 (median of 10.5 days postexposure; range = 10–11 days); specimens from these four close contacts tested negative.

Epidemiologic and clinical features of Omicron infection are still being described. Observations from this investigation, which included one patient who experienced reinfection^¶¶^ after having been fully vaccinated, four patients who experienced reinfection, and one who experienced their first infection, suggest a shorter incubation period and a clinical syndrome similar to or milder than that associated with previously described variants in persons who have been vaccinated or previously infected, and add to existing evidence suggesting an increased potential for reinfection.*** Whereas the median SARS-CoV-2 incubation period has been described as ≥5 days (*2*,*3*), and closer to 4 days for the SARS-CoV-2 B.1.617.2 (Delta) variant,^†††^ the median incubation period^§§§^ observed in this cluster was approximately 3 days. Although few clinical descriptions of Omicron infections are available, mild illness among vaccinated patients has been reported (*4*). It is unknown whether the mild clinical syndromes or differing symptom descriptions are a result of existing immunity or altered clinical features associated with Omicron infection. The five reinfections, including one after full vaccination, might be explained by waning immunity, the potential for partial immune evasion by Omicron, or both. Conclusions drawn from these observations are limited by small sample size. More data will be needed to fully understand the epidemiology of the Omicron variant.

Travel history of the index patient and phylogenetic analysis of the secondary cases indicate an international introduction of the Omicron variant, consistent with other early cases identified in the United States (*1*). The recent emergence of Omicron, which is now projected to be the dominant variant in the United States,^¶¶¶^ reinforces the importance of vaccination, in coordination with other prevention strategies (e.g., masking and physical distancing), to protect people from COVID-19, slow transmission, and reduce the likelihood of new variants emerging. In addition, the rapid identification and epidemiologic characterization of this cluster underscore the importance of robust and timely genomic surveillance to detect and respond to emerging SARS-CoV-2 variants of concern.

## Footnotes

* https://www.cdc.gov/coronavirus/2019-ncov/science/science-briefs/scientific-brief-omicron-variant.html

†
https://ndc.services.cdc.gov/case-definitions/coronavirus-disease-2019-2021/

§ 45 C.F.R. part 46, 21 C.F.R. part 56; 42 U.S.C. Sect. 241(d); 5 U.S.C. Sect. 552a; 44 U.S.C. Sect. 3501 et seq.

¶
https://www.cdc.gov/coronavirus/2019-ncov/your-health/quarantine-isolation.html#closecontact

**
https://www.cdc.gov/vaccines/covid-19/clinical-considerations/covid-19-vaccines-us.html#:~:text=A%20person%20is%20considered%20fully,for%20fully%20vaccinated%20people

††
https://www.cdc.gov/coronavirus/2019-ncov/need-extra-precautions/people-with-medical-conditions.html

§§ Abdominal pain, diarrhea, fatigue, headache, muscle aches, chest pressure, chills, subjective fever, cough, runny nose, sore throat, and dizziness.

¶¶ https://ndc.services.cdc.gov/case-definitions/coronavirus-disease-2019-2021/#:~:text=The%20following%20should%20be%20enumerated%20as%20a%20new%20case

*** https://www.medrxiv.org/content/10.1101/2021.11.11.21266068v2

††† https://www.medrxiv.org/content/10.1101/2021.07.07.21260122v2

§§§ Incubation period is defined as the interval from earliest possible exposure of household contacts to the index patient until symptom onset.

¶¶¶
https://covid.cdc.gov/covid-data-tracker/#variant-proportions
